# Non‐pharmacological Intervention for Dementia Care: A Pilot Study in Ghana

**DOI:** 10.1002/alz70860_096480

**Published:** 2025-12-23

**Authors:** Daniel Naawenkangua Abukuri

**Affiliations:** ^1^ University of Ghana, Accra, Greater Accra Region, Ghana

## Abstract

**Background:**

Non‐pharmacological interventions are increasingly recognized as the primary approach for reducing dementia symptoms, surpassing pharmaceutical therapies in best practices. This crossover design aimed to culturally adapt non‐pharmacological interventions for dementia management in Ghana.

**Method:**

Twenty older adults (aged 65 and older), diagnosed with dementia, were recruited from the Alzheimer's and Dementia‐SLH Ghana facility. Data collection included cognitive assessments, physical health measures, and participant feedback to assess intervention effectiveness. Over six weeks, interventions such as dancing, reminiscence therapy, traditional games, physical exercise, and intellectually stimulating activities were implemented. Using a crossover design with two groups, Phase 1 involved pretesting, followed by six weeks of intervention for Group 1 in Phase 2 and post‐testing. Phase 3 reversed interventions, Phase 4 included post‐testing, Phase 5 mirrored Phase 2, and Phase 6 had a three‐week period without interventions for both groups, followed by post‐testing.

**Result:**

The majority of participants were female (65%) and had a mean duration of dementia diagnosis of 3.2 years (SD = 1.6). Throughout the six‐ week intervention period, participants consistently engaged in the prescribed activities. Compliance rates were high, with an average attendance rate of 92% across all intervention sessions. Both Group 1 and Group 2 exhibited notable improvements in cognitive function following the six‐week intervention periods (Phases 2 and 3), as evidenced by a mean increase of 12 points (*p* < 0.001). Additionally, participants in both groups showed significant enhancements in physical health following the intervention phases (Phases 2 and 3), with an average increase in mobility of 25% (*p* < 0.005) and a 15% increase in strength (*p* < 0.01). Moreover, participant feedback highlighted the positive impact of the interventions on their overall well‐being. Qualitative responses indicated a sense of enjoyment, social connection, and cultural relevance experienced during the intervention sessions.

**Conclusion:**

Our pioneering study on culturally adapted non‐pharmacological interventions for dementia in Ghana demonstrated significant improvements in cognitive function, physical health, and well‐ being among elderly patients. High compliance resulted in notable enhancements, supporting the prioritization of customized non‐ pharmacological strategies as a primary approach to managing dementia.

